# Frameworks, Methodologies, and Tools for Evaluating Large Language Models in Digital Mental Health Interventions: Protocol for a Scoping Review

**DOI:** 10.2196/91677

**Published:** 2026-09-14

**Authors:** Antonio Salinas-Layana, Álvaro Jiménez-Molina, Daniela Lira, Félix Alberto Véliz-Montoya, Alexi Venegas, Nicolás Muñoz, Mario Chandía, Rigoberto Rojas, Vania Martínez

**Affiliations:** 1 Doctorado en Psicoterapia, Universidad de Chile y Pontificia Universidad Católica de Chile, Santiago, Región Metropolitana, Chile; 2 Nucleus to Improve the Mental Health of Adolescents and Youths (Imhay), Santiago, Región Metropolitana, Chile; 3 Facultad de Psicología y Humanidades, Universidad San Sebastián, Santiago, Región Metropolitana, Chile; 4 Center for Research and Action on Social Determination and Mental Health (CIADES), Santiago, Región Metropolitana, Chile; 5 Departamento de Ciencias de la Educación, Área de Psicología Social, Universidad de Burgos, Burgos, Castilla y León, Spain; 6 VeryMind, Santiago, Región Metropolitana, Chile; 7 Centro de Medicina Reproductiva y Desarrollo Integral del Adolescente (CEMERA), Facultad de Medicina, Universidad de Chile, Profesor Alberto Zañartu 1030, Independencia, Santiago, Región Metropolitana, 8380455, Chile, 56 229786484

**Keywords:** mental health, mental disorders, mental health services, telemedicine, therapy, computer assisted, artificial intelligence, AI, digital health, chatbot, large language models, scoping review

## Abstract

**Background:**

Digital mental health interventions (DMHIs) can help close persistent gaps in access to assessment, prevention, and treatment. Recent advances in generative AI, particularly large language models (LLMs), further expand this promise by enabling natural language understanding, personalization, and empathic interaction across assessment, support, and therapeutic contexts. However, significant evaluation challenges persist, including a lack of standardized constructs and validated instruments, which limit the comparability, reproducibility, and generalizability of the findings. No systematic synthesis currently documents the frameworks, methodologies, and tools used to evaluate LLMs in DMHIs, thereby hampering the development of a comprehensive evidence base to guide future evaluation efforts.

**Objective:**

This scoping review aims to systematically map and synthesize the available evidence on frameworks, methodologies, and tools used to evaluate LLMs applied to DMHIs. Specifically, it aims to identify the constructs assessed, the instruments used, and the evaluation procedures and stages addressed.

**Methods:**

Following the PRISMA-ScR (Preferred Reporting Items for Systematic Reviews and Meta-Analyses extension for Scoping Reviews) guidelines, this scoping review will search 5 electronic databases (PubMed, Scopus, Web of Science, IEEE Xplore, and ACM Digital Library) from January 1, 2019, to September 15, 2025. Eligibility criteria will encompass both empirical studies and theoretical proposals evaluating LLMs embedded within DMHIs. Studies limited to risk detection or decision support systems without an intervention component will be excluded. Data extraction will capture information on conceptual frameworks, methodological designs, evaluation procedures and tools, measured constructs, and other relevant contextual information.

**Results:**

The systematic search was conducted between September 1 and 15, 2025, yielding 4273 records across the 5 databases. After duplicate removal, of the 4273 records, 2980 (69.7%) remained for screening. A pilot screening exercise involving 4 independent reviewers achieved high interrater reliability (free-marginal Randolph κ=0.81), with 76% (19/25 of the pilot sample) unanimous agreement, indicating adequate calibration of selection criteria. These figures are interim process indicators rather than final review findings as title and abstract screening of the remaining records is currently underway.

**Conclusions:**

This scoping review is expected to provide one of the first systematic syntheses of frameworks, methodologies, and tools used to evaluate LLMs in DMHIs. By identifying prevailing patterns and gaps, the resulting evidence map is intended to serve as a practical reference for researchers, developers, and policymakers working toward a scientifically grounded, safe, ethical, and effective deployment of LLMs in mental health interventions.

## Introduction

Improving population mental health remains a major global challenge amid the rising prevalence of mental disorders, persistent treatment gaps, and unmet demand for psychological services [1]. In this context, digital mental health interventions (DMHIs) offer effective and scalable alternatives [2,3]. However, robust real-world evidence on digital mental health technologies remains limited, with persistent gaps in rigorous evaluation frameworks, standardized methodologies, and transparency in outcome reporting [4].

Recent advances in generative AI, particularly large language models (LLMs), extend this promise by enabling complex, individualized interventions at scale, surpassing earlier rule-based systems that relied on decision trees and keyword matching [5]. LLMs show strong performance on “theory-of-mind tasks” (eg, interpreting indirect requests, tracking false beliefs, and inferring intentions), at times approaching or exceeding human levels [6], and are often perceived as empathic conversational partners [7]. These features enable the sensitive detection of shifts in mental state and the delivery of interactions tailored to individual needs [8,9]. Preliminary effectiveness evidence is encouraging: a recent randomized clinical trial of the therapeutic agent Therabot reported significant reductions in anxiety and depressive symptoms in a clinical population [10]. However, the overall strength and reliability of this emerging evidence base warrant cautious interpretation as many recent AI chatbot trials for anxiety and depression are characterized by small and demographically narrow samples; suboptimal or inappropriate control conditions; and heterogeneous, nonstandardized outcome measures that limit both the generalizability of their findings and the reproducibility of their procedures [11].

As LLMs are increasingly integrated into DMHIs, it is essential to understand and systematize their evaluation process. This imperative is underscored by ethical challenges such as responsiveness to risk scenarios [12,13]. The scoping review by Hua et al [8] identified substantive limitations such as the lack of standardized constructs and scales and the resulting proliferation of ad hoc instruments with uncertain validity and reliability, which hampers cross-study comparisons and weakens the evidence base. The literature also overemphasizes user experience constructs (eg, usability, accessibility, and perceived usefulness) at the expense of critical dimensions such as safety, privacy, and equity. In contrast, other constructs such as transparency, resilience, accountability, and explainability remain largely unexplored. Complementing this, the scoping review by Jin et al [14] on LLM applications in mental health systematized the most commonly used performance metrics (*F*_1_-score, precision, accuracy, and recall) to evaluate interactions both among LLMs and between LLMs and human professionals. While these metrics provide a quantitative foundation, the analysis highlighted important gaps: the limitations of LLMs in addressing complex clinical tasks and the need to balance operational efficiency with potential risks. The aforementioned review also underscored the risk-benefit trade-off, highlighting 3 sensitive domains: data privacy, model bias, and the ethical implications of clinical implementation [14].

While these recent scoping reviews have provided foundational insights into the use of LLMs in mental health, they remain insufficient to address the specific objectives of the present study. The review by Hua et al [8] primarily categorizes what evaluation constructs are measured across broad generative applications (including clinical assistants and diagnostic aids for health care providers) rather than systematically examining the conceptual frameworks, evaluation methodologies, and specific tools used exclusively within direct DMHIs. Similarly, the focus by Jin et al [14] on computational task performance metrics (eg, *F*_1_-score and precision) is insufficient to capture the comprehensive methodological approaches required to evaluate clinical interventions, which must also account for therapeutic appropriateness, clinical safety, and user-centered outcomes through structured clinical tools.

Therefore, to the best of our knowledge, no systematic synthesis currently documents the conceptual frameworks, methodologies, and tools used or proposed to evaluate LLMs within mental health interventions. This hampers understanding of the field, the identification of best practices, and the detection of evidence gaps, thereby constraining the development of rigorous and evidence-informed evaluation approaches. A scoping review is therefore an appropriate approach as it maps the breadth, nature, and characteristics of the available literature, including empirical studies and theoretical proposals, and provides an integrated overview of the state of the field [15]. Unlike effectiveness-focused systematic reviews, scoping reviews are well suited to emerging, methodologically heterogeneous areas. While they do not involve formal critical appraisal, they record reported limitations during data extraction [16].

In this way, this protocol structures a scoping review to answer the following question: what evidence exists regarding the frameworks, methodologies, and tools used to evaluate LLMs applied to DMHIs?

The following complementary questions are posed: (1) what constructs have been measured and with which instruments? (2) Which evaluation phases have been addressed?

The objective of the scoping review is, therefore, to systematize the evidence regarding the evaluation processes of LLMs aimed at DMHIs, identifying the frameworks, methodologies, and tools used.

For this purpose, a framework will be understood as a conceptual structure that organizes and defines which dimensions to evaluate and how they are related without specifying a particular procedure for carrying out the evaluation [17]. Methodology will be understood as the procedures carried out to conduct the evaluation [18]. For this review, methodology will include 3 core components: study design, measured constructs, and the type of analysis. Finally, a tool will be understood as a concrete resource used in evaluation, such as questionnaires, interviews, and chat session transcripts, among others.

For this protocol, DMHIs are defined as programs, applications, chatbots, virtual agents, or digital platforms designed to deliver therapeutic, preventive, or psychosocial support components through digital technologies with or without direct clinical supervision. Accordingly, DMHIs include applications, chatbots, virtual agents, or platforms that provide mental health intervention, but tools intended exclusively for decision support, data analysis, or risk identification without an intervention component are excluded. LLMs are understood as computational AI models trained on large volumes of textual data that use deep architectures (such as transformers) and contain billions of parameters, enabling them to identify complex language patterns and generate coherent responses to a wide variety of natural language processing tasks [19].

To define and identify the evaluation phases, the framework proposed by Ding et al [20] for conversational agents with AI in health interventions will be used. This framework proposes 4 sequential stages aligned with progression from initial testing to large-scale implementation: feasibility and usability, efficacy, effectiveness, and implementation.

## Methods

### Sources of Information

The PubMed, Scopus, Web of Science, IEEE Xplore, and ACM Digital Library databases will be consulted to ensure a comprehensive and balanced coverage of biomedical, multidisciplinary, and technological research. The search period will span January 1, 2019, to September 15, 2025. This time frame was selected because the transformer architecture, which underpins LLMs, was introduced in 2017 [21], and GPT-2, one of the first widely available LLMs, was released in 2019. The latter marked a turning point by enabling the widespread development and application of LLMs in various domains, including digital health [22].

To capture emerging developments and early-stage proposals, preprints indexed in the Web of Science Preprint Citation Index will be included. All search activities will be recorded and documented to ensure transparency and reproducibility.

### Eligibility Criteria

The inclusion and exclusion criteria were defined to ensure the relevance of the studies identified in the review. The population, concept, and context framework recommended by the Joanna Briggs Institute (JBI) [16] will be used as a guiding criterion primarily focusing on the concept and context domains given that, based on the objective of this scoping review, the population category is not informative. The concept refers to the frameworks, methodologies, and tools used to evaluate LLMs in DMHIs. The context encompasses laboratory and real-world scenarios as well as theoretical assessment proposals without establishing geographical or cultural restrictions to broadly capture the diversity of approaches.

Research published in recognized databases and recent gray literature will be included without language restrictions to capture both established and emerging production. On the other hand, works with a general focus on AI without explicit reference to LLMs, those in which the models do not incorporate a mental health intervention component, applications limited to data analysis or administrative tasks, and literature reviews will be excluded. Details on the criteria are provided in Textbox 1.

Textbox 1. **Inclusion and exclusion criteria.**

**Inclusion criteria**

- Empirical studies that evaluate large language models (LLMs) in the context of digital mental health interventions (DMHIs)
- Theoretical proposals for evaluating LLMs in DMHIs, including conceptual frameworks, methodological approaches, or evaluation tools
- Studies published in the PubMed, Scopus, Web of Science, IEEE Xplore, and ACM Digital Library databases from January 1, 2019, to September 15, 2025
- Preprint gray literature studies available in the Web of Science Preprint Citation Index
- Studies published in any language if an English-language abstract is available for screening

**Exclusion criteria**

- Studies that use broad terms such as “AI” or “generative AI” but do not specifically refer to the use of an LLM in the intervention
- Studies in which the LLM does not incorporate a specific component intended for mental health interventions (a specific component is understood to mean, eg, providing emotional support, generating dialogue for therapeutic purposes, or providing behavior change strategies focused on mental health)
- Studies in which the LLM is used for data analysis, diagnosis, or administrative support without an intervention component
- Studies that are literature reviews, such as systematic reviews or scoping reviews

To improve screening consistency, eligibility criteria will be operationalized using 2 complementary decision rules applied during study selection. First, studies will be required to explicitly report or propose methods for evaluating LLM-based systems, including empirical evaluations using predefined criteria (eg, safety, usability, and clinical relevance) or theoretical contributions such as structured frameworks, methodologies, or tools for assessment. Studies lacking an explicit evaluative component will be excluded. Second, studies will be required to involve LLM-based systems within the DMHI context as defined in the population, concept, and context framework. Systems without an evaluative focus or without an intervention-oriented function will be excluded.

To further delimit boundary cases, the following worked examples operationalize the eligibility criteria:

- Included—an LLM-based chatbot delivering therapeutic dialogue, emotional support, or behavior change strategies that are evaluated against defined criteria (eg, safety, usability, or clinical relevance) and a conceptual framework, methodology, or tool proposed to evaluate LLM-based DMHIs
- Excluded—a chatbot described only at the design or architecture level without any evaluation, an LLM used solely for suicide risk detection or triage without support or an intervention delivered to the user, an LLM clinical decision support tool assisting clinicians rather than delivering an intervention to end users, and a study referring to “AI” or “generative AI” without specifying the use of an LLM in the intervention

Ambiguous or borderline cases will be resolved through discussion among reviewers, with arbitration by a senior reviewer when consensus is not reached.

### Search Strategy

The search strategy was designed in a replicable manner through an iterative process within the research team based on previous exploratory searches conducted by the team and a published protocol [23]. An initial set of key terms and Boolean operators was developed for each database in relation to three main concepts: (1) assessment, metrics, or frameworks; (2) LLMs; and (3) DMHIs. After an initial search process, the “NOT” operator was incorporated to reduce irrelevant records (eg, unrelated health conditions and review articles) following iterative testing conducted while minimizing the risk of excluding relevant studies within the scope of the review.

The complete strategy used for Web of Science can be found in Multimedia Appendix 1. It served as a template for adapting searches across the other platforms, with the necessary modifications applied to maximize the identification of relevant studies. The search was conducted in all predefined databases without language restrictions. The period was restricted to publications from January 1, 2019, to September 15, 2025. Gray literature was searched through the Web of Science Preprint Citation Index and supplemented by identifying studies in the reference lists of included articles and previous reviews.

### Article Selection

The studies selected in the search will be reviewed to eliminate duplicates using 2 software programs. First, EndNote 2025 (Clarivate Analytics) will be used for an initial reference import and to remove duplicates. Subsequently, the data will be exported to Rayyan (Qatar Computing Research Institute), where a second duplicate check will be performed.

The study screening process will be carried out in 2 phases using the Rayyan platform. Before the formal screening process, a pilot title and abstract screening exercise was conducted on a random sample of 25 records to assess the feasibility of the eligibility criteria and calibrate reviewer agreement. Four independent reviewers applied the predefined inclusion and exclusion criteria, classifying each record as included, excluded, or potentially eligible. This calibration process was repeated until an agreement level of 75% or higher was achieved, consistent with previously established standards [16].

Following this pilot phase, the formal screening process will begin. In the first phase, title and abstract screening will be conducted using the full dataset. The remaining records will be divided into 3 blocks of equal size. One reviewer will screen all blocks, whereas the other 3 reviewers will independently screen 1 block each. All records will undergo double screening at the title and abstract level, with disagreements resolved through consensus with a senior researcher.

In the second phase, the full texts of the preselected studies will be evaluated for inclusion by pairs of reviewers consisting of a mental health professional and a technology professional. Disagreements will initially be resolved through discussion between the 2 reviewers. In cases in which consensus cannot be reached, a third evaluator, selected according to the nature of the disagreement (mental health or technology), will intervene and make the final decision. This procedure aims to ensure the reliability of the selection process and integrate complementary disciplinary perspectives that help minimize potential biases. Given the emerging maturity of the field and the objective of this review, a quality assessment of the articles will not be conducted. Articles in languages other than English or Spanish will be translated using GPT-5 (OpenAI).

The entire process of identification, screening, inclusion, and exclusion will be described narratively and illustrated using a flowchart in accordance with the PRISMA-ScR (Preferred Reporting Items for Systematic Reviews and Meta-Analyses extension for Scoping Reviews) recommendations in the final manuscript (Checklist 1).

### Data Extraction

A data charting form developed by the research team will be used, informed by the objectives of this scoping review, the JBI guidance for scoping reviews [16], and preliminary pilot-testing. The charting framework is structured to capture four main categories relevant to the review questions: (1) study and intervention characteristics, including publication details, population, and DMHI purpose; (2) LLM characteristics, including model name and version and accessibility characteristics; (3) evaluation characteristics, including reported evaluation frameworks, evaluation phase, evaluation target, evaluation approach and methodology, data source and sample, evaluation domains, measured constructs, indicators and metrics, assessment instruments or tools, and evaluation findings and outcomes; and (4) study limitations reported by the authors.

The charting framework will include a codebook providing operational definitions for each data extraction field, with examples where applicable. The codebook will specify that only information explicitly reported in the included studies will be extracted and that fields will be coded as “not reported” when relevant information is unavailable.

To improve consistency in the extraction of evaluation-related information, particularly given the expected heterogeneity in terminology across studies, an a priori set of evaluation domains and measured constructs informed by the existing literature [5,8,20] will be used as a procedural tool to guide data charting and standardize the identification and coding of reported assessment areas. This set is not intended as a fixed taxonomy or comprehensive classification system but rather as a pragmatic starting structure that supports consistent extraction and synthesis across studies. The set will remain iterative, allowing for the refinement of domains and constructs and the incorporation of additional categories identified inductively during the charting process.

Evaluation domains will provide a high-level structure for organizing and synthesizing broader areas of assessment reported in the literature, whereas measured constructs will capture the specific attributes, processes, or outcomes evaluated within each domain. The initial set of evaluation domains includes safety and risk response, clinical efficacy and effectiveness, usability, user experience and engagement, therapeutic interaction and relational quality, explainability and transparency, bias, fairness and equity, privacy and data governance, accountability and clinical governance, and technical performance and reliability. These domains reflect key dimensions of evaluation that are both commonly assessed and increasingly highlighted in the literature on AI-based interventions in health care, including areas that remain underrepresented but are considered important for comprehensive assessment. Each domain is defined in the codebook and paired with example constructs and indicators, as detailed in Multimedia Appendix 2. All modifications will be documented, including their rationale and timing, and will be reported in the final review manuscript.

The extraction will be conducted by 6 reviewers organized into 3 interdisciplinary pairs, with each pair consisting of one mental health expert and one technology expert. The process will unfold in 2 stages.

In the first stage, 3 studies will be randomly selected for pilot extraction. Each interdisciplinary pair will independently extract data from these studies, working collaboratively in real time using shared extraction forms. Following independent extraction by all 3 pairs, the whole team will convene to calibrate criteria, resolve inconsistencies, and ensure interdisciplinary alignment before proceeding to the main extraction phase.

In the second stage (main extraction), after calibration, the remaining studies will be divided into 3 equal blocks, with each block assigned to 1 of the 3 interdisciplinary pairs. Pairs will work collaboratively using shared extraction forms, with each expert completing domain-specific fields while maintaining ongoing dialogue to contextualize findings. To enhance accuracy and verification, the extraction process will incorporate human-AI collaboration [24] using NotebookLM (Google). NotebookLM will be used to support cross-checking of extracted information, verification of technical and clinical terminology, and identification of potential inconsistencies. However, all extracted data will undergo final human validation to ensure accuracy and interpretive rigor.

Discrepancies in data extraction will be resolved through discussion between the pair of reviewers. If a consensus cannot be reached, a third senior researcher will serve as the arbitrator. The software Zotero (Corporation for Digital Scholarship) will be used for bibliographic management, and the extraction process will be conducted in Google Workspace, facilitating synchronous work, change traceability, and transparent recording of modifications.

### Data Analysis and Presentation of Results

The analysis will follow a descriptive and thematic approach aimed at mapping frameworks, methodologies, and tools used for evaluating LLMs in DMHIs. In accordance with the guidelines for scoping reviews [16], the aim will not be to synthesize or assess the certainty of the results but rather to organize the evidence in a structured manner.

The synthesis will be based on the grouping of extracted data into predefined categories aligned with the research questions. Studies will be grouped according to evaluation frameworks, methodologies, and tools used, as well as by the constructs and instruments reported within each evaluation domain. In addition, evaluation phases will be used as an organizing category to describe how and when evaluation is conducted across studies.

Within each category, results will be summarized descriptively to identify how frequently specific frameworks, constructs, instruments, and phases are reported and how they are distributed across the literature. This will allow for a structured comparison of evaluation approaches without attempting to generate inferential or effect-based conclusions.

The findings will be presented using tables; narrative summaries; and, where relevant, diagrams or concept maps illustrating patterns and relationships. The presentation will follow an inductive approach and will be reported in accordance with the PRISMA-ScR guidelines.

### Protocol Registration

This scoping review protocol was registered in the Open Science Framework on November 10, 2025 (digital object identifier: 10.17605/OSF.IO/PAZYQ). The systematic database search was conducted between September 1 and 15, 2025, and a pilot title and abstract screening exercise was completed before protocol registration. These preliminary activities were conducted to assess the feasibility of the search strategy and refine the screening procedures. Registration was completed before the initiation of the formal screening phase and before any full-text assessment, data extraction, or evidence synthesis activities. The registration record includes the complete search strategy, eligibility criteria, screening and extraction procedures, and data extraction framework. Any amendments to the protocol will be documented with rationale and date in the Open Science Framework registration and transparently reported in the final manuscript in accordance with PRISMA-ScR and *JBI Manual for Evidence Synthesis* guidelines.

### Ethical Considerations

As this is a scoping review, participant recruitment does not apply, and ethics approval is not required.

## Results

The following results represent interim process indicators from the screening phase of the review. Article selection is still ongoing, and these findings should not be interpreted as final review outcomes.

**Figure 1.**
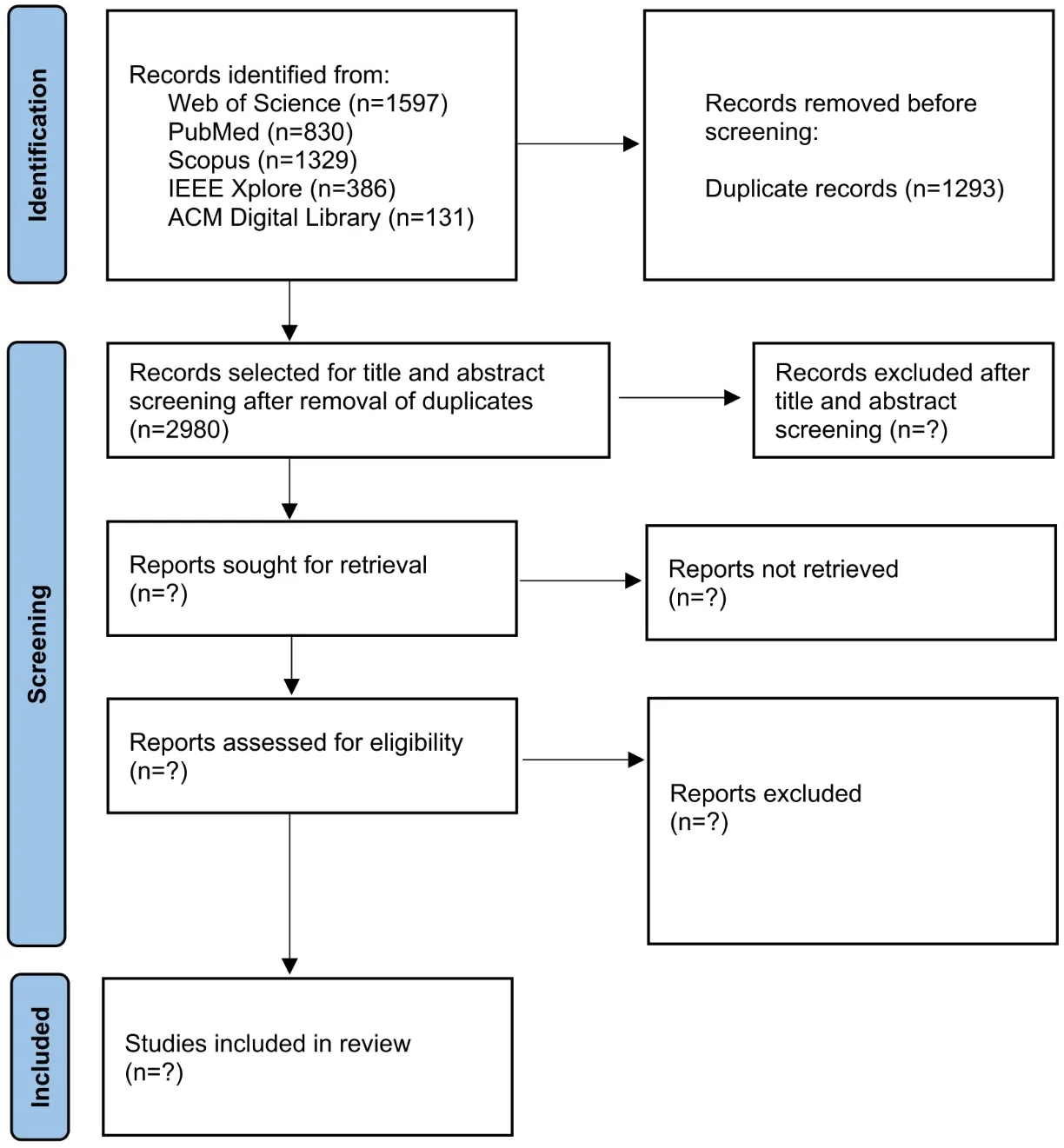
Preliminary PRISMA-ScR (Preferred Reporting Items for Systematic Reviews and Meta-Analyses extension for Scoping Reviews) flowchart.

The study was partially supported by research funding, a doctoral scholarship supporting the principal investigator’s doctoral training, and institutional support for a coauthor. Therefore, no single specific funding date applies to the study as a whole. Study conceptualization began in August 2025, and the systematic database search was conducted from September 1 to 15, 2025, across 5 bibliographic databases. At the time of submission, the review was in the data extraction phase, and data analysis had not yet begun. A total of 4273 records were identified from the following databases: 1597 (37.4%) from Web of Science (Core Collection and Preprint Citation Index), 830 (19.4%) from PubMed, 1329 (31.1%) from Scopus, 386 (9.0%) from IEEE Xplore, and 131 (3.1%) from ACM Digital Library. Of the 4273 records, after removing 1293 (30.3%) duplicates, 2980 (69.7%) remained for title and abstract screening (Figure 1).

A pilot selection test was carried out based on the screening of titles and abstracts. High interrater agreement was observed among evaluators, with a free-marginal Randolph κ coefficient [25] of 0.81 calculated in 3 nominal categories (0=no, 1=yes, and 2=maybe), indicating almost perfect agreement beyond chance. This coefficient was selected for this phase because it does not impose fixed marginal distributions and is appropriate when the prevalence of categories may be unbalanced among reviewers. Complementary descriptive metrics showed unanimous agreement of 76% (19/25 records in the pilot sample for which all 4 reviewers assigned the same eligibility category [included, excluded, or potentially eligible]) and mean pairwise agreement of 87.3% (131/150 pairwise record comparisons, SD 4.68%), supporting the procedural consistency of the selection criteria and thereby supporting the initiation of formal study selection.

The final results are expected to be prepared and submitted for publication in December 2026.

## Discussion

### Expected Findings

We anticipate that this review will provide a structured overview of the frameworks, methodologies, and tools used to evaluate LLMs in DMHIs, with particular attention to the constructs, instruments, and evaluation phases reported across studies. We expect to find a field still concentrated in early-stage feasibility and usability evaluations characterized by a predominance of ad hoc instruments over validated clinical measures; uneven coverage of the safety, privacy, equity, and accountability domains; and only emerging contributions addressing the more advanced efficacy, effectiveness, and implementation stages. A scoping review is the most appropriate methodological approach given the nascent and heterogeneous nature of the field, characterized by conceptual diversity and rapid technological expansion [8,26].

### Comparison With Prior Work

Existing reviews have begun to examine LLMs in mental health contexts. Hua et al [8] identified critical inconsistencies in evaluation methodologies, finding that most studies rely on ad hoc scales rather than validated clinical instruments and that safety, privacy, and algorithmic accountability remain largely unaddressed in the literature. Similarly, Jin et al [14] highlighted that LLMs should function as complementary tools rather than replacements for human clinicians and emphasized the absence of rigorous ethical and safety evaluation standards. However, these reviews did not primarily focus on mapping the evaluation frameworks and methodologies used to assess LLM-based DMHIs in diverse contexts. This scoping review aims to address this gap by systematically charting existing approaches and identifying areas where further methodological refinement may be needed.

### Strengths and Limitations

The protocol presents several methodological strengths, including strict adherence to PRISMA-ScR and JBI guidelines [15,16]; a comprehensive search strategy that spans biomedical, technological, and multidisciplinary databases; and the inclusion of gray literature to capture emerging developments. A key limitation is the potential incomplete capture of unindexed or non–English-language literature, which may affect the comprehensiveness of the evidence map. Additionally, given the rapid evolution of LLM technologies, some of the evidence, evaluation approaches, or practices identified in this review may change over time, which should be considered when interpreting the long-term relevance and applicability of the findings.

### Future Directions

By systematically mapping current evaluation practices, this review will provide an overview of how LLM-based DMHIs are currently being assessed and highlight areas where further research is needed. The findings may inform future work aimed at developing more comprehensive evaluation approaches that better capture the multifaceted and context-dependent nature of human-LLM interactions in mental health settings, including clinical, ethical, and user-centered dimensions.

Results will be disseminated through peer-reviewed publication, conference presentations, and a plain-language summary to facilitate access among relevant stakeholders.

### Conclusions

This scoping review is expected to provide one of the first systematic evidence syntheses of the frameworks, methodologies, and tools used to evaluate LLMs in DMHIs. By identifying prevailing patterns and gaps, the resulting evidence map is intended to serve as a practical reference for researchers, developers, and policymakers working toward a scientifically grounded, safe, ethical, and effective deployment of LLMs in mental health interventions.

## Supplementary material

10.2196/91677 Multimedia Appendix 1 Complete search strategy used in the Web of Science database.

10.2196/91677 Multimedia Appendix 2 Data extraction form.

10.2196/91677 Checklist 1 PRISMA-ScR checklist.

## Abbreviations

DMHI: digital mental health intervention
JBI: Joanna Briggs Institute
LLM: large language model
PRISMA-ScR: Preferred Reporting Items for Systematic Reviews and Meta-Analyses extension for Scoping Reviews
