# Supplementary material for: Frameworks, Methodologies, and Tools for Evaluating Large Language Models in Digital Mental Health Interventions: Protocol for a Scoping Review
Source: JMIR Res Protoc. 2026 Sep 14;15:e91677. doi: 10.2196/91677 (PMC13574329; doi:10.2196/91677)
Supplement: Supplementary file 1 [file resprot-v15-e91677-s001.docx]

Search date: September 15, 2025

Database: Web of Science Core Collection and Preprint Citation Index

Platform: Clarivate Web of Science

| Step | Query | Results |
| --- | --- | --- |
| #1 | TS=(evaluat* OR assess* OR validat* OR framework* OR metric* OR benchmark* OR "model evaluation" OR "Validation Study" OR "Randomized Controlled Trial" OR "exploratory stud*" OR pilot* OR "usability" OR "feasibility")  NOT TS=(diabetes OR dental OR cancer OR cardiovascular OR "literature review" OR "scoping review" OR "meta-analysis" OR "meta-analyses" OR "systematic review") | 16,839,503 |
| #2 | TS=("Large Language Models" OR "LLM" OR "LLMs" OR "chatbot" OR "Artificial Intelligence" OR "AI" OR "Generative Artificial Intelligence" OR "GenAI" OR "GAI" OR "language model*" OR "Natural Language Processing" OR "embedding" OR "LLaMA" OR "GPT" OR "ChatGPT" OR "BERT" OR "RoBERTa" OR "Transformer model" OR "BERTScore") | 1,591,439 |
| #3 | TS=("mental health intervention*" OR "mental health support" OR "mHealth" OR "psychological intervention*" OR "psychotherapy" OR "telepsychiatry" OR "DMHI" OR "DMH" OR "psychosocial support" OR "psychological treatment*" OR "mental health care" OR "support mental health" OR "Mental Healthcare" OR "Mental Health Literacy" OR "e-mental health") | 193,238 |
| #4 | #1 AND #2 AND #3 | 1,754 |
| #5 | #1 AND #2 AND #3 - 2019 - 2025 | 1,597 |
